# Case Report: Right aortic arch associated with an isolated left innominate artery

**DOI:** 10.3389/fped.2026.1809728

**Published:** 2026-04-20

**Authors:** Ronghui Zheng, Chaoyue Yan, Jiaguang Song, Mei Zhu

**Affiliations:** Department of Ultrasound, Shandong Provincial Hospital Affiliated to Shandong First Medical University, Jinan, Shandong, China

**Keywords:** case report, computed tomography angiography, echocardiography, isolated left innominate artery, right aortic arch

## Abstract

**Background:**

Right aortic arch encompasses multiple anatomical variants, among which the subtype involving an aberrant left subclavian artery is most frequently encountered. By contrast, a right aortic arch with an anomalous origin of the left brachiocephalic artery arising from the main pulmonary artery constitutes an exceptionally rare congenital vascular anomaly and is most often identified incidentally.

**Case presentation:**

A rare case of a right aortic arch accompanied by an isolated left innominate artery is described. The anomaly was initially identified during the fetal period, subsequently confirmed after birth, and monitored over time to inform decision-making regarding the most appropriate timing of surgical intervention.

**Conclusion:**

Despite its extreme rarity, this congenital anomaly is being recognized with increasing frequency due to technological advances and heightened awareness of fetal echocardiography. In the postnatal setting, the integration of computed tomography angiography with three-dimensional reconstruction facilitates more accurate and timely diagnosis. This multimodal approach supports early identification and management, thereby potentially reducing the risk of irreversible injury.

## Background

A right aortic arch is defined as an aortic arch that courses over the right bronchus and is embryologically derived from the right fourth pharyngeal arch artery in conjunction with the right dorsal aorta (1). Its prevalence is estimated at approximately 0.1% in the general population, while markedly higher rates, ranging from 13% to 34%, have been reported among patients with tetralogy of Fallot (2).

During the fetal period, right aortic arch is commonly classified into four principal subtypes: right aortic arch with an aberrant left subclavian artery (RAA-ALSA), right aortic arch with mirror-image branching, right aortic arch with an isolated left subclavian artery, and right aortic arch with an isolated left innominate artery (RAA-ILINA). Of these, RAA-ALSA is observed most frequently (3). Right aortic arch associated with the anomalous origin of the left brachiocephalic artery from the main pulmonary artery corresponds to the RAA-ILINA subtype and constitutes an exceptionally rare congenital vascular anomaly, which is typically identified incidentally (4). Echocardiography has become the preferred screening method due to its simplicity and safety, particularly for fetuses and infants. Its core imaging planes include the three-vessel-tracheal plane and the long-axis/coronary plane of the aortic arch in the suprasternal fossa, which typically allow early detection of abnormalities. CTA, as the gold standard, provides precise anatomical data for surgical planning. Surgical intervention is generally indicated when airway compression, esophageal compression, insufficient perfusion of the left upper limb/cerebral blood supply, or pulmonary hypertension caused by left-to-right shunting occur. The primary objectives of surgery include relieving vascular ring compression on the airway/esophagus, restoring continuity between the left brachiocephalic artery and aortic arch, and normalizing perfusion of the left common carotid artery and left subclavian artery to prevent steal syndrome. The surgical approach involves first releasing vascular rings in cases with vascular rings, followed by direct reconstruction of the left brachiocephalic artery in cases without vascular rings. Direct anastomosis is preferred, with thorough mobilization of the proximal left brachiocephalic artery and aortic arch lateral wall to achieve end-to-side anastomosis, thereby restoring normal perfusion.

## Case presentation

The patient was a neonate delivered by cesarean section at 38 weeks and 2 days of gestation to a primigravid mother (gravida 1, para 1). The mother had received routine prenatal care at another institution. Results of the oral glucose tolerance test were 4.96, 8.77, and 6.65 mmol/L. Blood pressure and thyroid function remained within normal limits throughout pregnancy, and there was no reported exposure to teratogenic or other harmful substances. Nuchal translucency measured 0.09 cm, and noninvasive prenatal testing indicated a low risk of chromosomal abnormalities.

**Figure 1:**
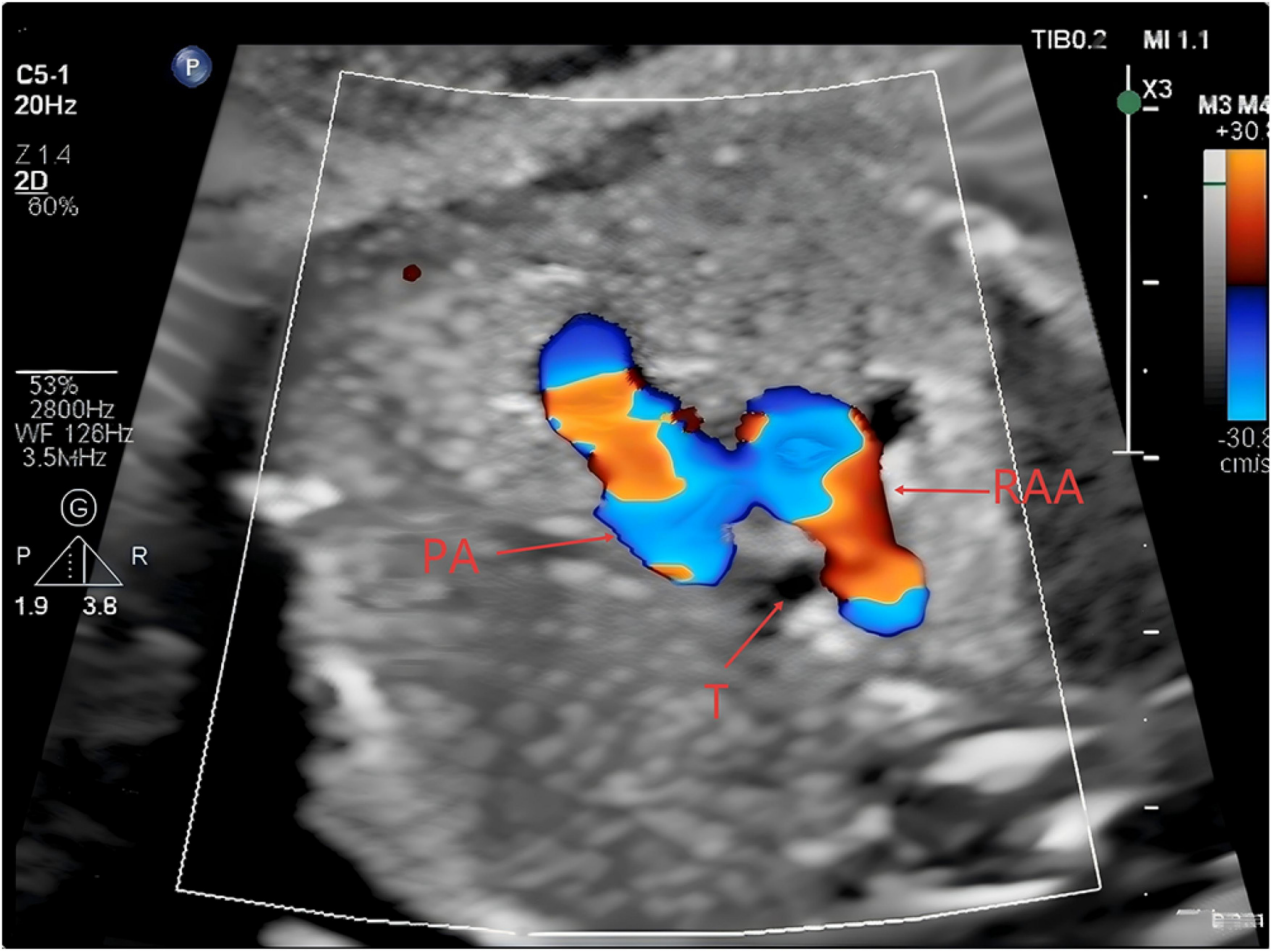
Aortic arch nnpositioned to the right side of the trachea (PA, pulmonary artery; RAA, right aortic arch; T, trachea).

**Figure 2:**
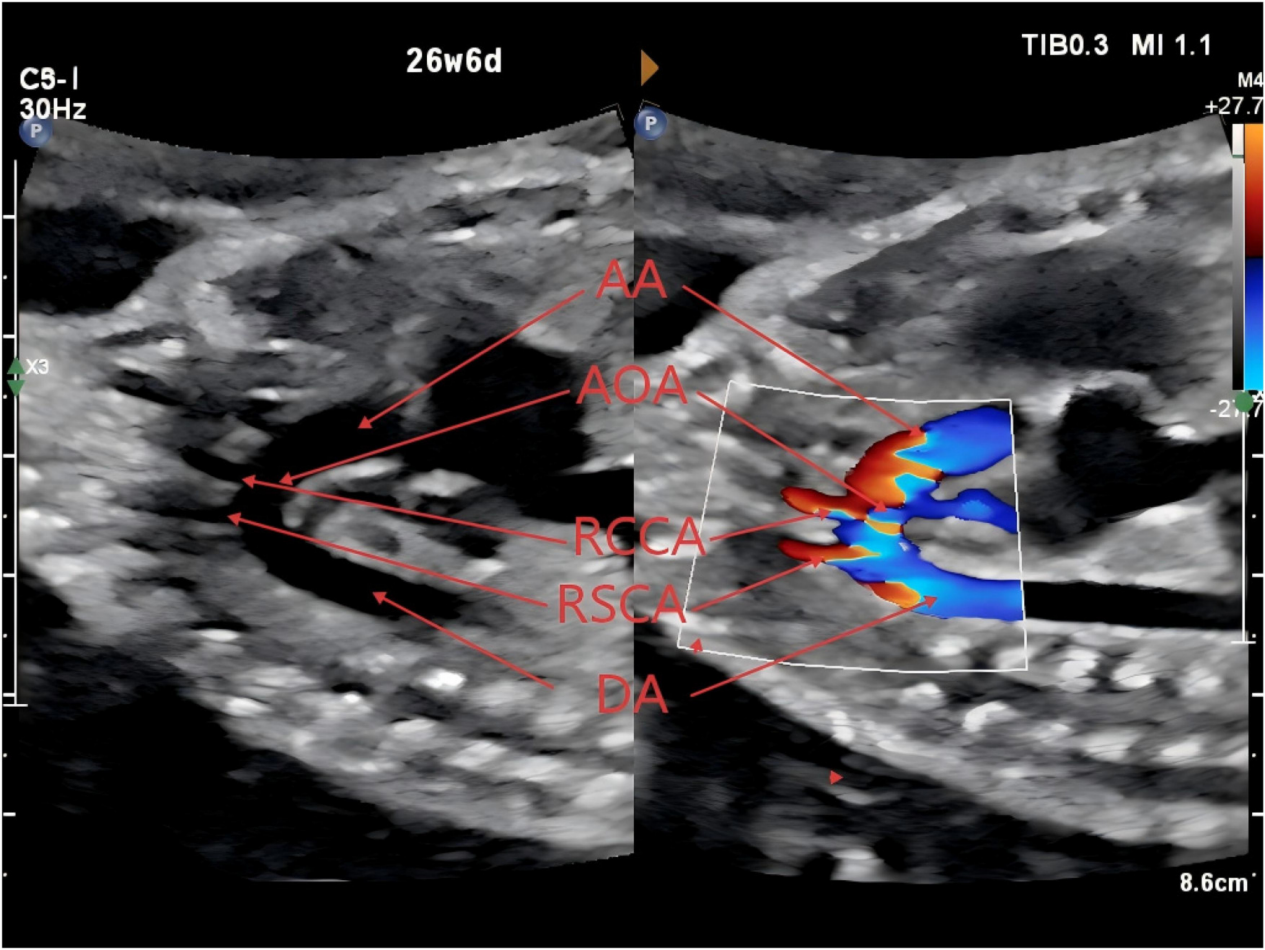
Right aortic arch giving rise sequentially to the right common carotid artery and the right subclavian artery (AA, aortic arch; AOA, aortic arch origin; RCCA, right common carotid artery; RSCA, right subclavian artery; DA, descending aorta).

**Figure 3:**
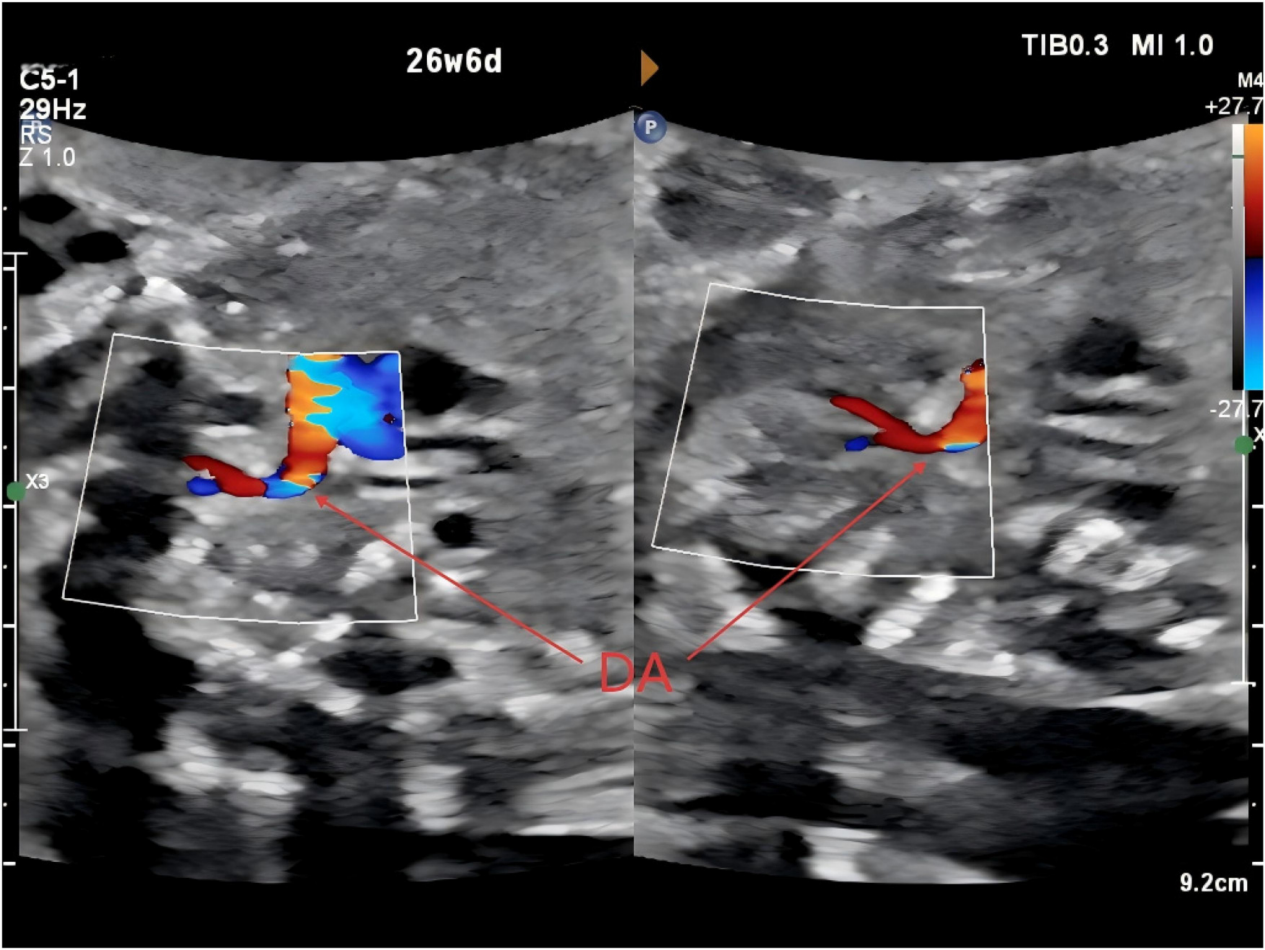
An anomalous branch arising from the pulmonary artery, consistent with a left ductus arteriosus (DA, ductus arteriosus).

**Figure 4:**
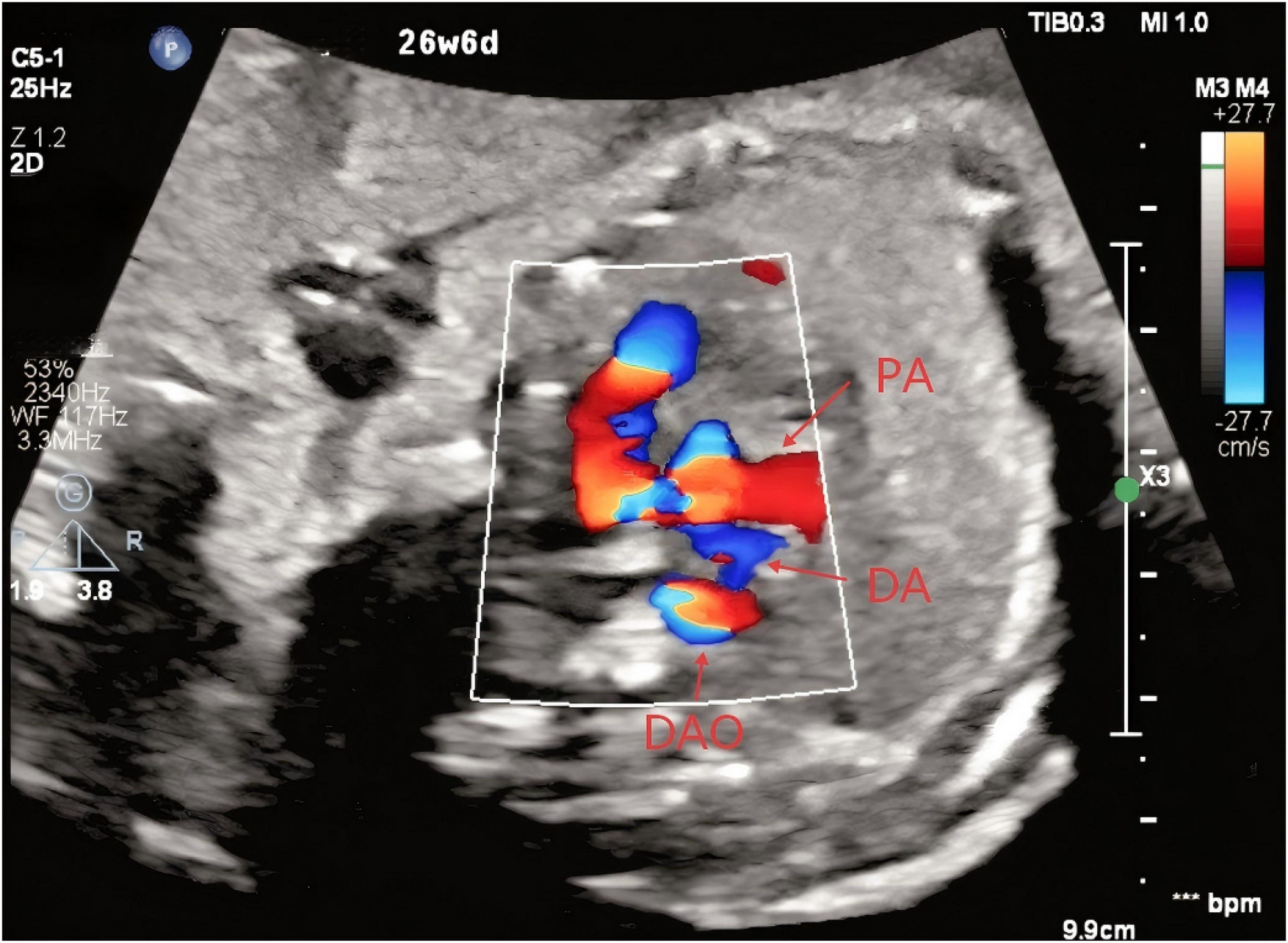
Right-sided ductus arteriosus connecting the pulmonary artery to the descending aorta (PA, pulmonary artery; DA, ductus arteriosus; DAO, descending aorta).

At 23 weeks of gestation, a fetal anomaly scan raised suspicion of a right-sided ductus arteriosus and a right aortic arch. Subsequent fetal echocardiography performed at 27 weeks of gestation at our institution demonstrated that the aortic arch coursed to the right of the trachea (Figure 1), giving rise sequentially to the right common carotid artery and the right subclavian artery (Figure 2). The left innominate artery was observed to originate from the pulmonary artery, presumably through a left ductus arteriosus (Figure 3), while the right ductus arteriosus exhibited a normal anatomical connection (Figure 4). Collectively, these findings were consistent with an aortic arch anomaly characterized by a right aortic arch, a right-sided ductus arteriosus, and anomalous origin of the left common carotid and left subclavian arteries from the pulmonary artery.

**Figure 5:**
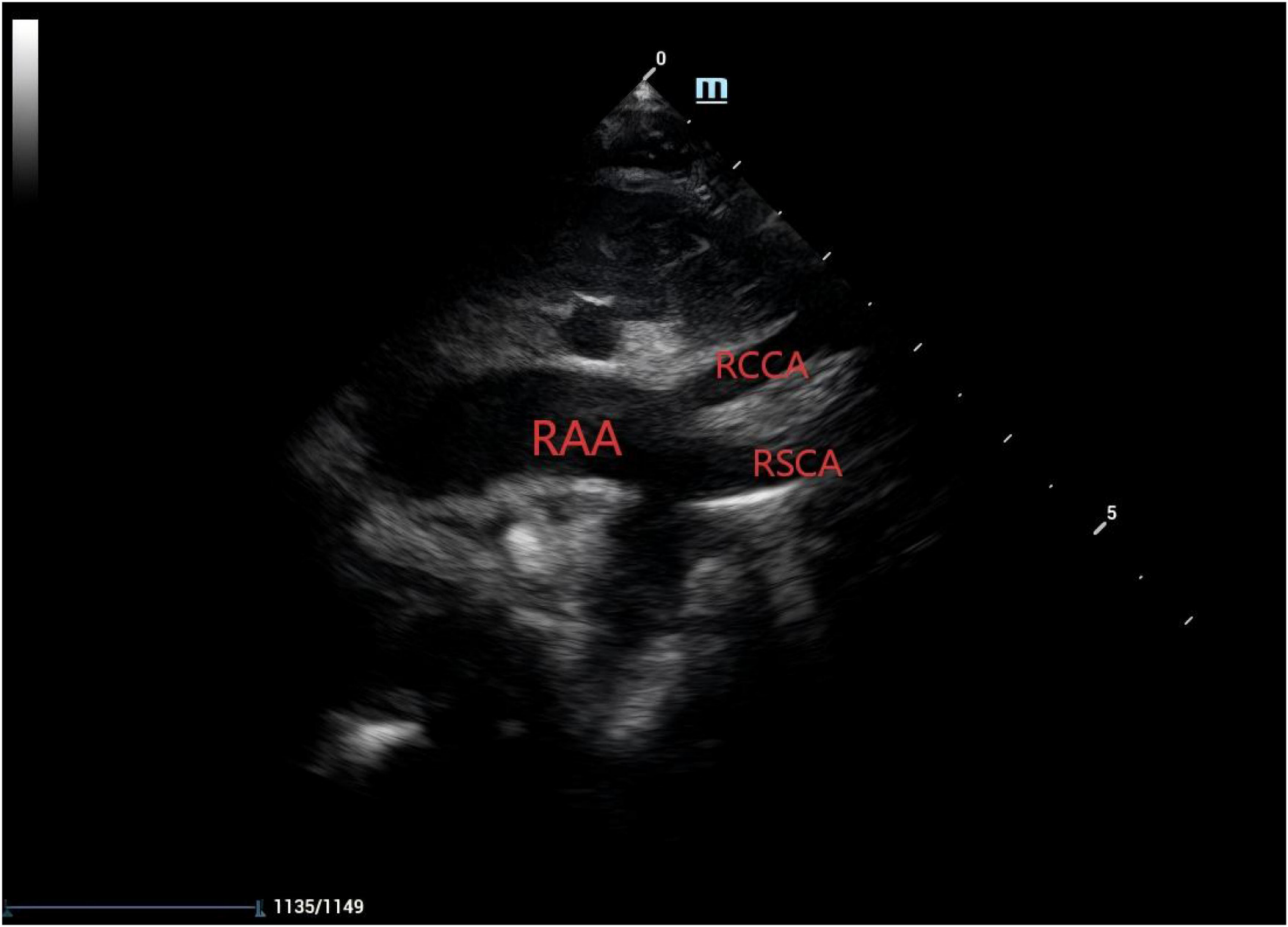
Postnatal echocardiographic view demonstrating two branches arising from the right aortic arch (RAA, right aortic arch; RCCA, right common carotid artery; RSCA, right subclavian artery).

Amniocentesis was not performed, and no targeted intervention was undertaken during pregnancy. Following an uncomplicated delivery, the neonate was admitted to the neonatal unit at our institution for clinical observation. Postnatal bedside echocardiography confirmed the presence of a right aortic arch, with the right common carotid artery and right subclavian artery arising directly from the aortic arch (Figure 5).

**Figure 6:**
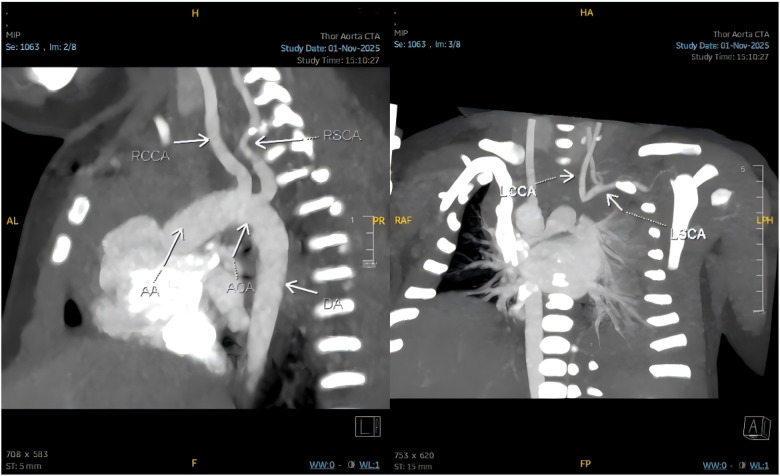
Postnatal closure of the left ductus arteriosus with formation of a blind-ended proximal segment of the left innominate artery (LBCA, left brachiocephalic artery).

The proximal segment of the left brachiocephalic artery terminated in a blind end, whereas the distal segment ascended cranially and subsequently bifurcated into the left common carotid artery and left subclavian artery (Figure 6). These imaging features were consistent with a right aortic arch accompanied by an isolated left innominate artery and a patent foramen ovale.

**Figure 7:**
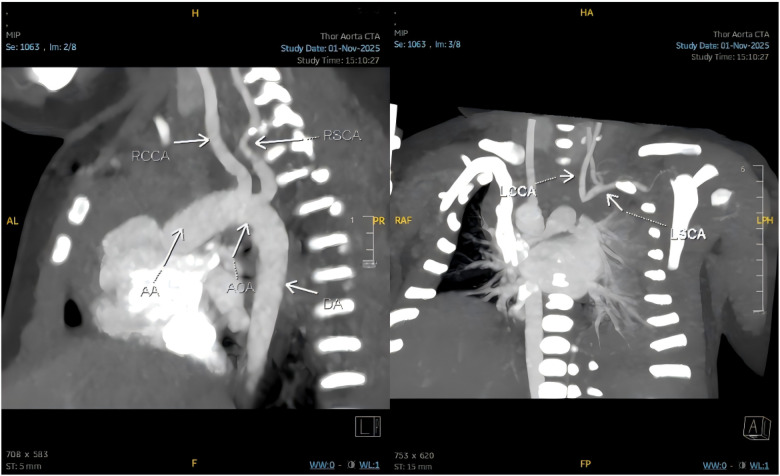
Chest computed tomography angiography confirming a right aortic arch with an isolated left innominate artery (RCCA, right common carotid artery; RSCA, right subclavian artery; AA, aortic arch; AOA, aortic arch origin; DA, descending aorta; LCCA, left common carotid artery; LSCA, left subclavian artery).

**Figure 8:**
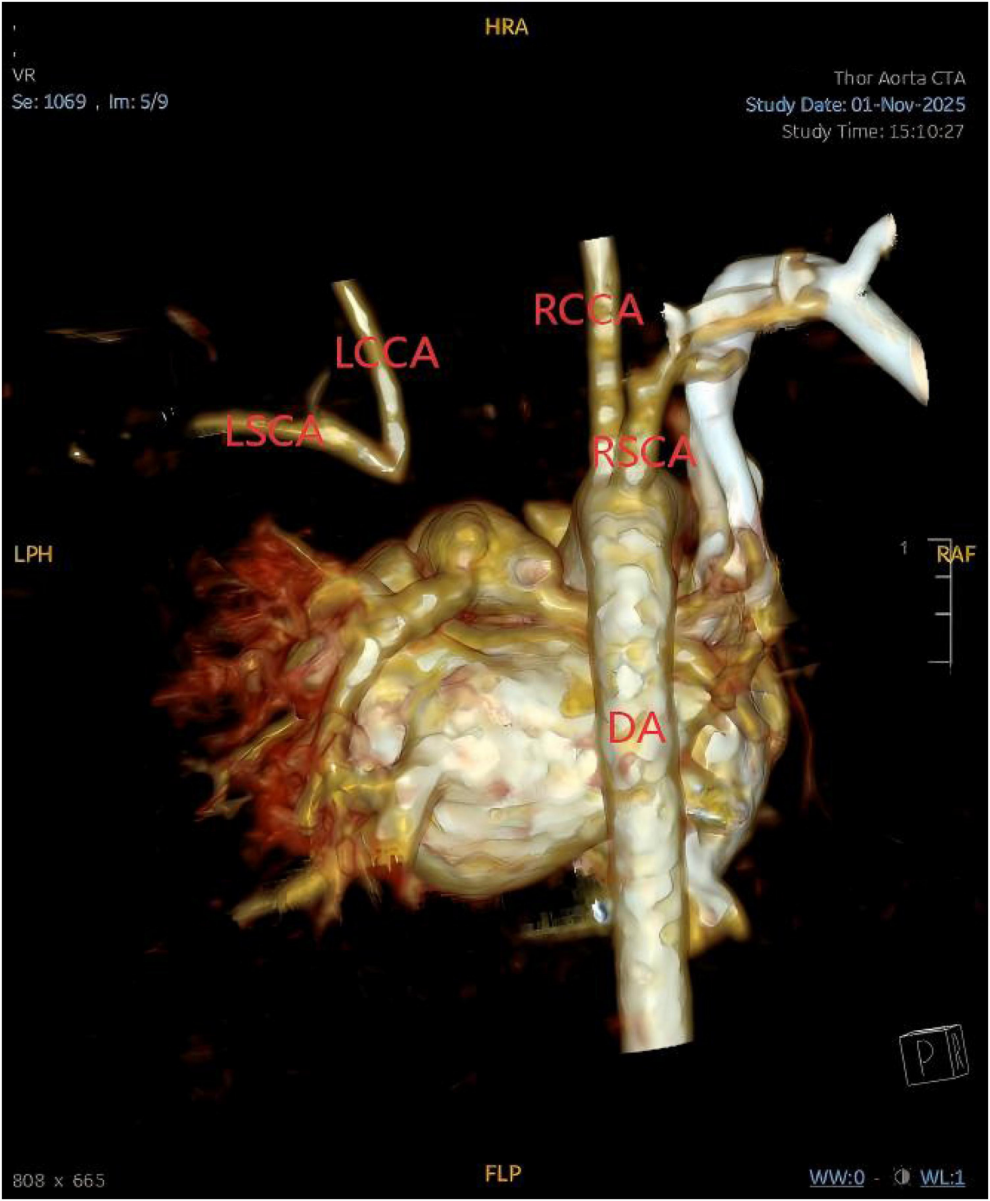
Three-dimensional reconstructed image illustrating the anatomical configuration of the isolated left innominate artery (LSCA, left subclavian artery; LCCA, left common carotid artery; RCCA, right common carotid artery; RSCA, right subclavian artery; DA, descending aorta).

Further evaluation with bedside vascular ultrasonography demonstrated an anomalous origin of the left common carotid artery, presumed to arise from the pulmonary artery. Hemodynamic assessment revealed a steal phenomenon involving the left common carotid and vertebral arteries, along with reduced flow velocity in the left subclavian artery. Chest computed tomography angiography confirmed the presence of a right aortic arch, patent foramen ovale, and an isolated left innominate artery (Figure 7), with subsequent three-dimensional reconstruction for detailed anatomical assessment (Figure 8).

Non-contrast brain magnetic resonance imaging demonstrated symmetric hyperintense signals in the bilateral globus pallidus on T1-weighted sequences, for which clinical correlation was recommended to exclude bilirubin encephalopathy. The cerebral hemispheric medulla exhibits slightly prolonged T1 and T2 signals, with isointense or slightly hypointense T2-FLAIR signals, and no significant abnormal signals observed on DWI. Susceptibility-weighted imaging showed no obvious abnormalities. The neonate remained in stable general condition and was discharged with plans for scheduled follow-up.

**Figure 9:**
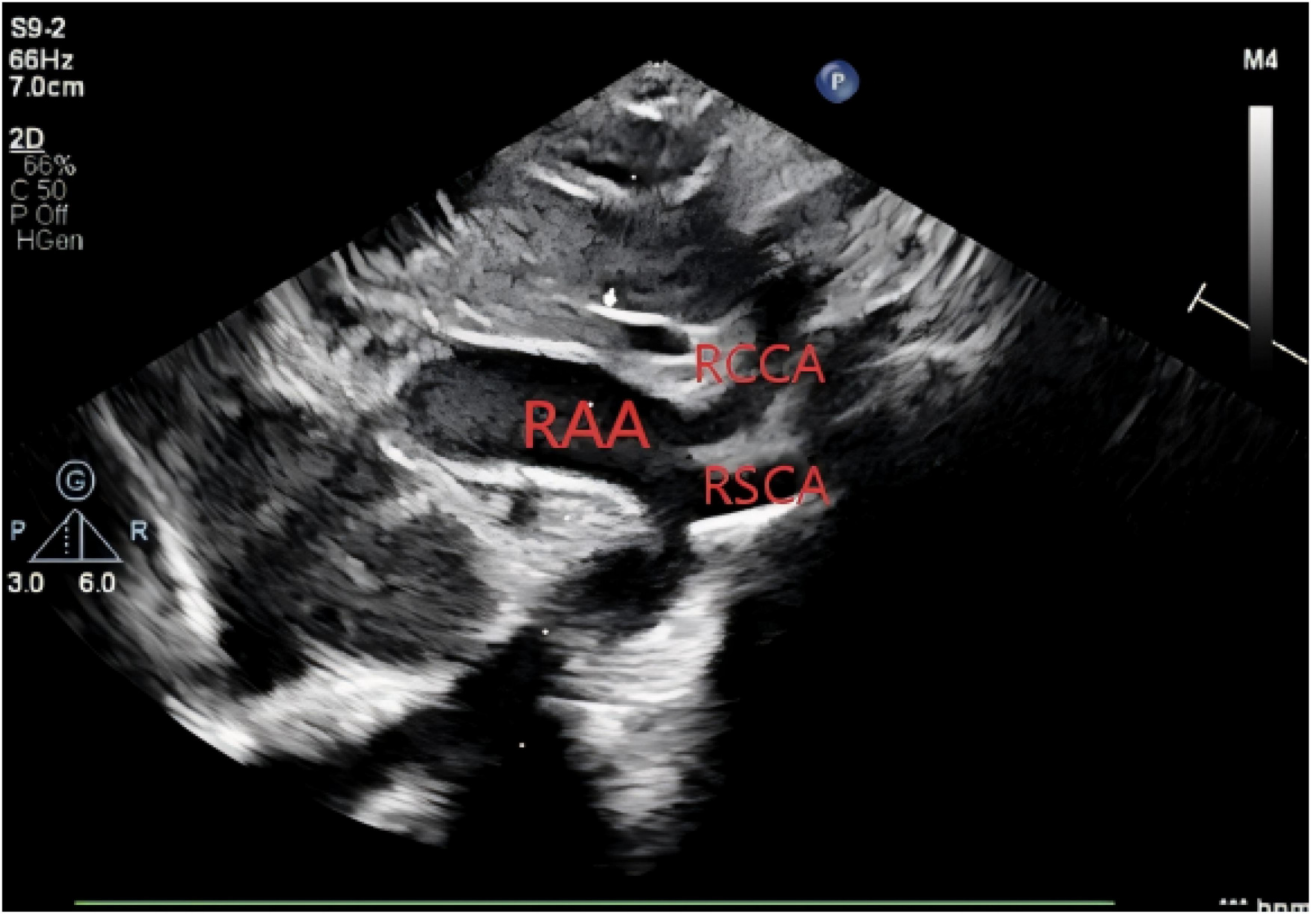
Echocardiographic findings at 1 month of age showing two branches arising from the right aortic arch (RAA, right aortic arch; RCCA, right common carotid artery; RSCA, right subclavian artery).

At 1 month of age, the patient underwent a follow-up assessment at our institution. Transthoracic echocardiography demonstrated a right aortic arch from which only the right common carotid artery and right subclavian artery originated, with no detectable brachiocephalic artery. Color Doppler flow imaging detected low-velocity retrograde flow within the left common carotid and left subclavian arteries. Collectively, these findings remained consistent with a diagnosis of right aortic arch associated with patent foramen ovale and an isolated left innominate artery (Figure 9).

## Discussion

In this patient, the left brachiocephalic artery was connected to the pulmonary artery through a left ductus arteriosus during the fetal period. Following birth, regression of the ductus arteriosus led to the isolation of the left brachiocephalic artery. A right aortic arch with an isolated left innominate artery represents an exceptionally rare congenital cardiovascular malformation. Affected patients may be diagnosed incidentally during prenatal evaluation or may present postnatally with clinical manifestations such as pulmonary hypertension, vertebrobasilar steal syndrome, left upper limb claudication, or post-exertional dizziness (5).

Edwards proposed a developmental hypothesis involving selective regression of specific segments of a double aortic arch to account for the spectrum of aortic arch anomalies (4). According to this concept, formation of an isolated artery results from regression at two distinct levels, causing separation of a vessel, such as the left subclavian artery or left brachiocephalic artery, from the aortic arch. The isolated artery subsequently maintains continuity with the pulmonary artery through derivatives of the distal sixth aortic arch, also referred to as the pulmonary arch (2).

When a right aortic arch is associated with a left ductus arteriosus and an aberrant left subclavian artery, a vascular ring may develop, with potential compression of the esophagus or trachea. In the present case, bilateral ductus arteriosi were identified during the fetal period; however, regression of the proximal aortic arch and distal descending aorta prevented the formation of a complete vascular ring. After birth, physiological closure of the ductus arteriosus occurred, and no esophageal compression or tracheal obstruction was observed (6, 7).

With ongoing advances in fetal echocardiographic techniques and a deeper understanding of fetal right aortic arch anatomy, the three-vessel view and the three-vessel–trachea view have become valuable tools for identifying the majority of associated anomalies (6, 8). During prenatal ultrasonography, detection of a right aortic arch in combination with a right-sided ductus arteriosus or bilateral ductus arteriosi warrants meticulous evaluation of the origins of the head and neck vessels from the aortic arch. Any discontinuity between these vessels and the aortic arch should prompt consideration of an isolated left subclavian artery or an isolated left brachiocephalic artery (9).

When evaluating an isolated left brachiocephalic artery, sagittal imaging typically demonstrates only two head and neck vessels arising from the aortic arch, namely the right common carotid artery and right subclavian artery arranged in an anteroposterior orientation, along with a left brachiocephalic artery originating from the main pulmonary artery. In contrast, an isolated left subclavian artery may be more easily overlooked, as three head and neck vessels can often be visualized arising from the aortic arch, namely the left common carotid artery, right common carotid artery, and right subclavian artery from anterior to posterior.

Further diagnostic clarification requires assessment of the spectral Doppler waveforms of the left subclavian and left common carotid arteries. Because peak flow velocity is highly dependent on fetal physiological conditions and cannot be measured simultaneously in paired vessels, direct comparison is challenging. From a theoretical standpoint, flow velocities in an isolated left subclavian artery or left common carotid artery are expected to be lower than those in the corresponding right-sided vessels (10, 11).

In this patient, the patent ductus arteriosus closed after birth; retrograde blood flow from the affected vertebrobasilar system into the subclavian artery resulted in subclavian steal syndrome. As the child grows and develops, if symptoms worsen and affect the development of the left brain tissue or upper limb, surgical intervention should be considered. During this period, cranial MRI can be used to detect any ischemic damage to brain tissue or growth restrictions, and differences in blood pressure, pulse, and oxygen saturation between the child’s upper limbs can be assessed to determine whether there is insufficient blood supply to the left upper limb. Surgical reconstruction of the blood supply to the isolated left innominate artery is the curative treatment for this condition. However, since the child is still growing and developing and shows no obvious clinical symptoms or developmental delays caused by steal syndrome, the decision was made to proceed with follow-up observation. The patient’s parents have a thorough understanding of the condition and agree that follow-up observation is appropriate at this stage. They believe that the timing of surgery should be determined by taking into account the child’s overall growth and development, the severity of shunt symptoms, and the growth of the great arteries and their branches.

The prognosis of fetal right aortic arch is closely dependent on the presence and severity of associated intracardiac and extracardiac malformations, as well as underlying chromosomal abnormalities. Chromosomal abnormalities are reported more frequently when intracardiac defects coexist, underscoring the importance of thorough evaluation for additional structural abnormalities (12, 13). In the present patient, no concomitant intracardiac or extracardiac malformations were identified, chromosomal analysis was not performed, and the overall clinical condition has remained stable. On this basis, the patient is being managed with continued follow-up.

However, vascular ultrasonography demonstrated a steal phenomenon involving the left common carotid and vertebral arteries. These findings highlight the need to consider the anatomical configuration and potential clinical benefits when determining the optimal timing for possible revascularization.

## Conclusion

Although this congenital anomaly is exceptionally rare, ongoing technological advancements and increased clinical awareness have contributed to higher detection rates during fetal echocardiography. These observations underscore the importance of systematically assessing the origins of the head and neck vessels whenever abnormalities in the position or number of the aortic arch or ductal arch are identified on prenatal imaging. Such a strategy may enhance prenatal diagnostic accuracy, and atypical spectral Doppler patterns of the common carotid or subclavian arteries warrant careful attention.

In the postnatal period, the combined use of computed tomography angiography and three-dimensional reconstruction allows for more precise and timely anatomical delineation. This integrated imaging approach supports early identification and management, which may help reduce the risk of irreversible injury.

## Data Availability

The datasets presented in this article are not readily available because of ethical and privacy restrictions. Requests to access the datasets should be directed to the corresponding author.
